# Supplementary material for: More than Just Host Plant Preferences for the Two Main Vectors of Xylella fastidiosa in Europe: Two Insect Species and Two Different Behaviors
Source: Insects. 2025 Apr 15;16(4):416. doi: 10.3390/insects16040416 (PMC12027782; doi:10.3390/insects16040416)
Supplement: Supplementary file 1 [file insects-16-00416-s001.zip › insects-3454203-supplementary.pdf]

Detailed Statistical Analyses

Differences in oviposition rates between the two vector species (**Vector**: *Neophilaenus campestris* or *Philaenus spumarius*), the three host plant species (**Plant**: *Calendula arvensis*, *Festuca arundinacea*, or *Medicago sativa*), and the two simulated scenarios of host diversity (**Host Diversity**: presence of a single host species vs. presence of multiple host species) over the study period were analyzed using a generalized mixed model with a negative binomial error distribution. The **number of eggs** laid per female was used as the dependent variable, with **Vector**, **Plant**, and **Host Diversity** as explanatory variables. All interactions among these variables were included in the model. **Temporal replicate** and **Bugdorm** were incorporated as random factors, while **Pot** (individual plants) was included as nested random factor within **Bugdorm**.

For each vector species, we assessed adult female oviposition preferences (**Place**: plant substrate or dry soil substrate) and how this preference varied with host plant species (**Plant**) and host diversity (**Host Diversity**) using generalized mixed models with a negative binomial error distribution. The **number of eggs** laid per female was the dependent variable, while **Place**, **Plant**, and **Host Diversity** were included as fixed explanatory factors. The interactions among these variables were also tested. **Temporal replicate**, **Bugdorm**, and **Pot** were initially included as random factors, but **Bugdorm** and **Pot** were later removed from the final models as they did not contribute to the covariance structure.

To examine host plant preference differences between monocotyledonous and dicotyledonous plant species for *P. spumarius* and *N. campestris* nymphs during their developmental period, generalized mixed models were used. The **number of nymphs** (counts) observed on each host plant species at each sampling day served as the dependent variable, with a Poisson error distribution specified for the model. Host plant species (**Plant**) was included as a fixed factor, and **Bugdorm** and **Pot** (nested within **Bugdorm**) were included as random effects. Sampling day (**Time**) was modeled as a repeated measure with an autoregressive (AR(1)) covariance structure. The interaction between **Time** and **Plant** was included to evaluate whether host plant preference changed over time.

For each observation time, differences in host plant preference (**Plant**: *M. sativa* vs. *F. arundinacea*) by *P. spumarius* or *N. campestris* were analyzed using exact goodness-of-fit tests. The **number of nymphs** observed on each plant species was compared to a theoretical 1:1 distribution (indicating no preference). Significant deviations from this expected distribution indicated a preference for one of the host plants.

To evaluate differences in nymphal developmental time between the two vector species when developing on their preferred hosts (*F. arundinacea* for *N. campestris*; and *C. arvensis* and *M. sativa* for *P. spumarius*), a generalized mixed model with a Poisson error distribution was used. **Developmental time** (time to adulthood) was the dependent variable, and **Vector** was included as a fixed explanatory variable. **Bugdorm** was included as a random factor.

To investigate whether *P. spumarius* nymphal developmental time differed among host plant species, a generalized mixed model with a Poisson error distribution was employed. **Developmental time** (time to adulthood) was the dependent variable, and **Plant** was the fixed explanatory factor. **Bugdorm** was included as a random factor.

Differences in survival rates between *P. spumarius* and *N. campestris* throughout their nymphal developmental stages, when developing on their preferred hosts (*F. arundinacea* for *N. campestris*; and *C. arvensis* and *M. sativa* for *P. spumarius*), were assessed using a generalized mixed model with a binomial error distribution. **Survival rate** (number of nymphs reaching adulthood divided by the initial number of nymphs) was the dependent variable, and **Vector** was the fixed explanatory factor. **Bugdorm** was included as a random factor.

To examine potential differences in *P. spumarius* nymphal survival rates when developing on different host plant species (*C. arvensis*, *M. sativa*, or *Prunus dulcis*), a generalized mixed model with a binomial error distribution was applied. **Survival rate** (number of nymphs reaching adulthood divided by the initial number of nymphs) was the dependent variable, and **Plant** was the fixed explanatory factor. **Bugdorm** was included as a random factor.
